# Effect of Global Climate Change Concern on Environmental Sensitivity of Nursing Students

**DOI:** 10.1111/phn.13434

**Published:** 2024-10-07

**Authors:** Dijle Ayar, Burak Gözüaçık, Bahar Şen

**Affiliations:** ^1^ Faculty of Health Science Pediatric Nursing Department Alanya Alaaddin Keykubat University Alanya Turkey; ^2^ Faculty of Health Science Department of Nursing Alanya Alaaddin Keykubat University Alanya Turkey

**Keywords:** environmental sensitivity, global climate change, nursing student

## Abstract

**Objective:**

This study was conducted to evaluate the effect of global climate change concern on environmental sensitivity in nursing students.

**Design:**

This study was descriptive, cross‐sectional, and correlational.

**Sample:**

The study was conducted with undergraduate nursing students (*n* = 350).

**Method:**

Descriptive Information Form, Climate Change Worry Scale, and Environmental Sensitivity Scale were used as data collection tools in the study. The relationship between global climate change concern and environmental sensitivity of nursing students was evaluated by Pearson correlation analysis, and the effect of climate change concern on environmental sensitivity of nursing students was evaluated by simple regression analysis.

**Results:**

It was determined that there was a positive and moderately significant relationship between the mean total scores of the Climate Change Concern Scale and the mean total scores of the Environmental Sensitivity Scale (*r* = 0.388, *p* < 0.001).

**Conclusions:**

As a result of this study, it was found that as nursing students' concern about global climate change increased, their sensitivity toward the environment also increased.

## 1 Background

Living organisms and nonliving entities coexist and interact in our environment and human beings play the most important role in this interaction (Yesil and Turan 2020). In today's world, environmental problems, which continue to increase with an acceleration parallel to developing technology, industrialization and rapid population growth, and whose main source is human beings, have reached significant dimensions, and situations such as unconscious use of resources, global warming, ozone depletion, and greenhouse effect cause environmental problems to increase gradually (Aytop, Çetinkaya, and Tulan 2021).

It is stated that the environmental sensitivity of individuals is important in preventing environmental problems, environmental sensitivity enables individuals to have a strong sense of responsibility toward the environment and protect the environment by developing environmental awareness (Ibrahim et al. 2021; Bala et al. 2023). “Environmental sensitivity,” which is the way environmental problems are perceived by society, is defined as the ability of individuals who make up the society to be aware of and fulfill their duties to the environment (Yesil and Turan 2020; Aytop, Çetinkaya, and Tulan 2021; Bala et al. 2023). Environmentally sensitive individuals are those who are aware of environmental pollution and population growth, protect natural resources, and are willing to have positive attitudes toward environmental problems. It is recommended that environmental awareness should be spread to all segments of society regardless of age, culture, and education level, and that each individual should make an individual contribution to a healthy and clean environment within his/her own life cycle, therefore, it is recommended to increase the studies that will increase the environmental awareness of the society and affect environmental awareness (Aytop, Çetinkaya, and Tulan 2021; Bulut and Kasap 2023; Yesil and Turan 2020). In the studies, it is emphasized that people can live in a healthier and safer environment by developing environmental awareness or determining the factors affecting it. Among the factors affecting environmental sensitivity, it is stated that individuals' concerns about climate change may also be effective.

Today, climate change, which directly or indirectly affects all countries of the world, has become one of the most important global problems (Şimsek 2020). Climate change is the biggest health problem of the 21st century and threatens the society we live in from all aspects (WHO 2023). Turkey, which is one of the countries located in the region with high vulnerability in terms of climate change, is shown among the countries in the risk group in terms of the possible impacts of climate change (Şen and Özer 2018; Turan 2018; OECD 2019; Demirbaş and Aydın 2020).

Although climate change has various impacts, especially its impacts on human health are high and serious. Studies show that climate change will cause the distribution of some infectious disease vectors (lice, fleas, ticks, flies, etc.) to change, decrease in water resources and shrinkage of agricultural areas, change in the seasonal distribution of some allergic pollen species, and increase in deaths due to heat waves. It is emphasized that exposure to these changes may have negative effects on human health (Kahraman and Senol 2018; WHO 2023; IPCC 2023). One of the most important effects of global climate change is its psychosocial effects on individuals. Studies indicate that extreme weather events (drought, flood, forest fire, etc.) can cause negative emotions such as post‐traumatic stress disorder, anxiety, substance abuse, sleep disorders, trauma, and anxiety in individuals (Cianconi, Betrò, and Janiri 2020; Cankardaş and Sofuoğlu 2021; IPCC 2023). It is emphasized that especially climate change concerns (extreme weather events and uncertainties about the future in the ecosystem, etc.) trigger fear and anxiety in people, and degraded natural environments can cause stress in individuals (Stewart 2021). The fact that environmental problems reach dangerous situations and threaten public health significantly requires all health professionals, especially nurses, to be sensitive to the environment. Especially university education is of great importance in raising environmentally sensitive, protective, and educative individuals. In order to educate students who are well‐equipped about the environment and thus to bring individuals with positive attitudes toward the environment into society, it is extremely important to determine environmental sensitivity and influencing factors at all levels of education and to make necessary plans in this direction (Luque‐Alcaraz et al. 2024).

In this context, this study was carried out in order to examine the effect of the level of concern about individual climate change on the environmental sensitivity of nursing students, who are in constant contact with healthy and sick individuals both in the field and in the hospital environment, who assume an important role in raising awareness and education of the society, and to create this awareness in nursing students before they step into the profession.

### 1.1 Research Questions

Does the concern of nursing students about global climate change worry scale anxiety sub‐dimension have an impact on their environmental sensitivity?

Does the concern of nursing students about climate change worry scale feeling of helplessness have an impact on their environmental sensitivity?

## 2 Methods

### 2.1 Research Design

This descriptive, cross‐sectional, and correlational study was conducted to evaluate the effect of global climate change concern on environmental sensitivity in nursing students.

### 2.2 Participants

The study was carried out between December 2022 and November 2023 with undergraduate nursing students (*n* = 350) studying at Alanya Alaaddin Keykubat University, Faculty of Health Sciences, Department of Nursing, attending the 1st, 2nd, 3rd, and 4th grades. In order to determine the sample size, the required sample size was determined as 125 based on the correlation analysis with medium effect size (0.15) by accepting Type 1 error 0.01 and Type 2 error 0.01 (99% power) in the G‐Power program. However, in order to clearly understand the relationship between the variables, 350 nursing students who met the inclusion criteria and agreed to participate in the study, and had a written consent form were included in the study. The criteria for inclusion in the sample were determined as: (a) being a nursing department student and (b) students who voluntarily accepted to participate in the study and had a consent form. The exclusion criteria from the sample were: Students who did not voluntarily agree to participate in the study/students who initially agreed to participate in the study but later wanted to leave the study for any reason. Students were informed about the research purpose, and written consent was obtained from those who agreed to participate. A total of 410 nursing students were given questionnaires, but 60 students did not want to fill them out. In addition, identity information was not requested from the students while completing the questionnaires.

### 2.3 Measures

“Descriptive Information Form,” “Climate Change Worry Scale,” and “Environmental Sensitivity Scale” were used as data collection tools in the study.

#### 2.3.1 Descriptive Information Form

The descriptive information form was developed by reviewing the literature (Stewart 2021; Gezer and İlhan 2021; Yeşil and Turan, 2020; Tümer, İpek, and Ercan 2024). It consists of 11 questions about the socio‐demographic characteristics of the students, including age, gender, class, parental education level, whether they have undergraduate courses on the environment and global climate change, or whether they have taken courses on these issues.

#### 2.3.2 Climate Change Worry Scale

The original scale was developed by Stewart (2021). The Turkish validity and reliability study of the scale was conducted by Gezer and İlhan (2021) in order to determine the level of worry about climate change in individuals since the observed results of climate change or its possible future effects cause psychological reactions such as fear, stress, trauma, depression, and anxiety in individuals. The Turkish scale consists of two sub‐dimensions and 10 items. The first sub‐dimension is “anxiety” sub‐dimension and the second sub‐dimension is “feeling of helplessness” sub‐dimension. The total explanation percentage of the two‐factor structure is 64.17. The total Cronbach's alpha coefficient of the scale is 0.91 and the sub‐dimension Cronbach's alpha values are 0.87 and 0.83, respectively. The item‐total score correlation coefficient of the scale is between 0.56 and 0.74 and the factor loading values are between 0.61 and 0.75. The climate change worry scale has a maximum score of 50. The scale is a valid and reliable measurement tool that can assess climate change concerns in individuals and university students (Gezer and İlhan 2021). In this study, the total Cronbach's alpha coefficient of the scale was found to be 0.912.

#### 2.3.3 Environmental Sensitivity Scale

The scale developed by Yeşil and Turan (2020) to measure attitudes toward the environment is a 5‐point Likert type and consists of a total of 20 items. The total explanation percentage of the scale is 67.293%. The KMO value of the scale is 0.845. The total Cronbach's alpha value of the scale is 0.845. According to the confirmatory factor analysis results, the fit values of the scale are as follows: *X* ^2^ = 286.535; Df = 160; *X* ^2^/df = 1.791; GFI = 0.869; AGFI = 0.828; NFI = 0.853; RFI = 0.826; IFI = 0.930; TLI = 0.915; CFI = 0.928; SRMR = 0.057; RMSEA = 0.068. The environmental sensitivity scale has a maximum score of 100. The Environmental Sensitivity Scale was found to be a valid and reliable tool for measuring attitudes toward the environment (Yesil and Turan 2020). In this study, the total Cronbach's alpha coefficient of the scale was found to be 0.927.

#### 2.3.4 Ethics Approval

Permission to use the scales was obtained by e‐mail from the researchers who performed the Turkish validity and reliability of the scales used in the study. In order to conduct the study, written permission was obtained from Alanya Alaaddin Keykubat University Non‐Invasive Clinical Research Evaluation Commission and Alanya Alaaddin Keykubat University Faculty of Health Sciences, where the study would be conducted, and written permission was obtained from the students participating in the study on a voluntary basis by explaining the purpose of the study and using the “informed consent form.” Students were informed about the research purpose, and written consent was obtained from those who agreed to participate. Since individual rights should be protected in the study, the Helsinki Declaration of Human Rights was adhered to during the study period.

#### 2.3.5 Analytic Strategy

Descriptive data of nursing students were evaluated by number, percentage, and mean analyses. Whether the data fit a normal distribution was analyzed by calculating the kurtosis and skewness coefficients (Kurtosis and skewness coefficients of data were found between ±2). The relationship between global climate change concern and environmental sensitivity of nursing students was evaluated by Pearson correlation analysis, and the effect of climate change concern on environmental sensitivity of nursing students was evaluated by simple regression analysis. Whether the scale scores can be included in the regression model will be evaluated by multiple linear correlation analysis for regression. Tolerance, variance inflation factor (VIF), and condition index values were used to determine which of the independent variables would be included in the model (to determine the existence of multicollinearity). Independent variables with a VIF value of <10, tolerance value of >0.2, and condition index value of <15 were included in the regression analysis. The significance level of 0.05 was considered acceptable.

## 3 Results

The study was carried out with undergraduate nursing students (*n* = 350) studying at Alanya Alaaddin Keykubat University, Faculty of Health Sciences, Department of Nursing, attending the 1st, 2nd, 3rd, and 4th grades. The mean age of the students included in the study was 20.90 ± 2.5 years (min: 17; max: 42) and 37.4% (*n* = 131) were male students. When the grade level of the students was analyzed, 42.6% (*n* = 149) were in the first grade, 17.4% (*n* = 61) were in the second grade, 21.4% (*n* = 75) were in the third grade, and 18.6% (*n* = 65) were in the fourth grade. Descriptive information about nursing students is given in Table 1.

**TABLE 1:** Descriptive characteristics of nursing students.

Descriptive characteristic	*n*	%
Gender		
Male	131	37.4
Female	219	62.6
Grade		
1st grade	149	42.6
2nd grade	61	17.4
3rd grade	75	21.4
4th grade	65	18.6
Mother's education status		
Literate	21	6.0
Primary school	141	40.3
Secondary school	66	18.9
High school graduate	74	21.1
University (undergraduate)	38	10.9
Postgraduate	10	2.9
Father's education level		
Literate	16	4.6
Primary school	98	28.0
Secondary school	91	26.0
High school graduate	98	28.0
University (undergraduate)	47	13.4
Income status		
Income less than expenditure	97	27.7
Income equal to expenditure	226	64.6
Income more than expenditure	27	7.7
Have you taken a course/seminar on environmental education?		
Yes	64	18.3
No	286	81.7
Have you taken a course/seminar on climate change?		
Yes	44	12.6
No	306	87.4

The mean total scores of the nursing students were found to be 37.73 + 8.84 for the Climate Change Worry Scale and 82.23 + 11.81 for the Environmental Sensitivity Scale (Table 2).

**TABLE 2:** Mean total scale scores of nursing students.

	X	SD	Minimum	Maximum
Climate Change Worry Scale	37.73	8.84	10	50
Environmental Sensitivity Scale	82.23	11.81	20	100

When the correlation between the mean total scores of the Climate Change Worry Scale anxiety sub‐dimension and the mean total scores of the Environmental Sensitivity Scale of nursing students was examined, it was determined that there was a positive and moderately significant relationship between the first‐dimension total scores of the Climate Change Worry Scale and the mean total scores of the Environmental Sensitivity Scale (*r* = 0.342, *p* < 0.001). When the correlation between the mean total scores of the Climate Change Worry Scale feeling of helplessness sub‐dimension and the mean total scores of the Environmental Sensitivity Scale of nursing students was examined, it was determined that there was a positive and moderately significant relationship between the second‐dimension total scores of the Climate Change Worry Scale and the mean total scores of the Environmental Sensitivity Scale (*r* = 0.295, *p* < 0.001). When the correlation between the mean total scores of the Climate Change Worry Scale and the mean total scores of the Environmental Sensitivity Scale of nursing students was examined, it was determined that there was a positive and moderately significant relationship between the mean total scores of the Climate Change Worry Scale and the mean total scores of the Environmental Sensitivity Scale (*r* = 0.388, *p* < 0.001) (Table 3).

**TABLE 3:** Correlation between climate change worry and environmental sensitivity of nursing students (*n* = 350).

	1	2	3
	*r*		
1. Mean Total Score of Environmental Sensitivity Scale	1.0		
2. Mean Total Score of Climate Change Worry Scale first‐dimension	0.342	1.0	
3. Mean Total Score of Climate Change Worry Scale second dimension	0.295		1.0

Abbreviation: *r*, correlation coefficient.

^*^ *p* < 0.001.

Three models were created by considering the relationships between the study variables (the mean total scores of the Climate Change Worry Scale anxiety sub‐dimension, the mean total scores of the Climate Change Worry Scale the feeling of helplessness sub‐dimension, and the mean total scores of the Environmental Sensitivity Scale) and environmental sensitivity.

According to Model 1, it was found that the regression model studied to examine the relationship between the mean total scores of the Climate Change Worry Scale anxiety sub‐dimension and Environmental Sensitivity of Nursing Students was significant (*F* = 46.023, *p* < 0.001). In Model 1, it was found that there was a positive and moderately significant relationship between the mean total score of the Climate Change Worry Scale anxiety sub‐dimension and the mean total score of the Environmental Sensitivity Scale of nursing students (*β* = 0.342, *p* < 0.001), and 11% of the factors affecting the environmental sensitivity of the students was explained by the mean total score of the Climate Change Worry Scale anxiety sub‐dimension of nursing students (Table 4).

**TABLE 4:** Predicting environmental sensitivity of nursing students' climate change worry (*n* = 350).

	Model 1	Model 2	Model 3
	*β*		
Climate Change Worry Scale anxiety sub‐dimension	0.342^*^		
Climate Change Worry Scale feeling of helplessness sub‐dimension		0.295^*^	
Climate Change Worry Scale			0.388^*^
*R*	0.342	0.295	0.388
*R* ^2^	0.117	0.087	0.150
*F*	46.023	33.260	61.625
SE	2.318	3.490	2.238
*B*	67.037	70.325	65.273
*t*	28.924	32.865	29.167
*p*	<0.001	<0.001	<0.001
DW	1.762	1.764	1.824

Abbreviations: *B*, standardized beta; DW, Durbin Watson; *R*, coefficient of common correlation; SE, standard error.

* *p* < 0.001

According to Model 2, it was found that the regression model studied to examine the relationship between the mean total scores of the Climate Change Worry Scale feeling of helplessness and Environmental Sensitivity of Nursing Students was significant (*F* = 33.260, *p* < 0.001). In Model 2, it was found that there was a positive and moderately significant relationship between the mean total score of the Climate Change Worry Scale feeling of helplessness sub‐dimension and the mean total score of the Environmental Sensitivity Scale of nursing students (*β* = 0.342, *p* < 0.001), and 8% of the factors affecting the environmental sensitivity of the students was explained by the mean total score of the Climate Change Worry Scale feeling of helplessness of nursing students (Table 4).

According to Model 3, it was found that the regression model studied to examine the relationship between climate change worry and environmental sensitivity of nursing students was significant (*F* = 61.625, *p* < 0.001). In the model, it was found that there was a positive and moderately significant relationship between the mean total score of the Climate Change Worry Scale and the mean total score of the Environmental Sensitivity Scale of nursing students (*β* = 0.388, *p* < 0.001), and 39% of the factors affecting the environmental sensitivity of the students were explained by the mean total score of the Climate Change Worry Scale of nursing students (Table 4).

## 4 Discussion

With the findings of this study, it was determined that there was a significant relationship between global climate change worry and environmental sensitivity of undergraduate nursing students and global climate change worry predicted environmental sensitivity. In this study, three models were created by considering the relationship between the variables. In Model 3, the relationship between global climate change worry and environmental sensitivity total mean scores of undergraduate nursing students was examined. It was determined that nursing students with a high mean score of global climate change worry also had a high mean score of environmental sensitivity, and global climate change worry had an important place among the factors affecting nursing students' environmental sensitivity (39%, Table 4). It was found that the mean total score of environmental sensitivity was 0.39 times higher for students with high global climate change worry than for students with low global climate change worry.

According to IPCC (2023) report, climate change is one of the most important problems of the world today and directly affects human health. Climate change can lead to respiratory diseases, food insecurity and consumption of unhealthy foods, malnutrition, and obesity, and consequently to various physical health problems such as diabetes and cancer (Kahraman and Senol 2018; WHO 2023). In addition, the observed consequences of climate change or the possible effects of climate change in the future negatively affect mental health by causing reactions such as fear, stress, trauma, depression, and anxiety in individuals (Innocenti et al. 2023). Extreme weather events and uncertainties about the future in the ecosystem trigger fear and anxiety in people, while degraded natural environments can stress individuals (Luque‐Alcaraz et al. 2024). Therefore, the problem of climate change that harms human health is the issue that all health professionals should focus on. In this context, nurses, whose professional professionalism is human, health, disease, and environment, have an important position on climate change. ICN (2018) emphasizes that nurses can take an active role in mitigating climate change and its effects, in this context, the subject of climate change should be included in the curriculum in the education of nursing students, students should want to learn about this issue during their education, and they should have positive attitudes toward this concept in the future (ICN 2018; Lopez‐Medina et al. 2019; Çakar 2023).

In the literature, it is emphasized that concern about climate change can trigger people's positive behaviors to reduce the negative consequences of climate change, and it is important how much individuals are concerned about climate change (Bouman et al. 2020). For this reason, it is stated that individuals' concerns about climate change are important to understand their belief that the effects of climate change will be eliminated, their willingness to take part in this process, and their sensitivity toward the environment (Brown, White, and Nicholas 2022; Fertelli 2023).

Universities have important responsibilities in raising individuals who will contribute to the improvement of the quality of life of the global community; who have the necessary knowledge, skills, and values; and who have developed environmental awareness. Today, when the effectiveness and importance of preventive health services are highly understood and felt, nursing is one of the most important professional groups to provide this service to healthy and sick individuals (Gök and Fırat Kılıç 2021). Nursing education is a professional education that responds to environmental problems and raises awareness and consciousness in nursing students by addressing global environmental problems in nursing practice. Throughout the history of nursing, nurses have been aware that the environment has an important place in patient care. Although environmental protection and environmental awareness are very important, in this context, it is necessary to provide this awareness and consciousness to nursing students in the period when they have not yet stepped into the profession and to train them in an equipped manner (Fırat Kılıç et al. 2024). In this direction, it is very important to determine the environmental sensitivity of nurse candidates who will provide preventive health services such as environment, environmental health, and counseling in the future and the factors affecting them (Gök and Fırat Kılıç 2021; Yılmaz Uzelli and Eşer 2021). In this study, it was determined that nursing students mostly did not receive training on global climate and environmental sensitivity in their education, and their knowledge on the subject was limited to what they learned from mass media.

In studies similar to our study, there are studies showing that individuals who are concerned about climate change also have high environmental sensitivity, that these two concepts affect each other, and that individuals taking action on climate change or reflecting it on their behavior are related to environmental sensitivity (van der Linden et al. 2017; Şen and Özer 2018). In the study conducted by Şen and Özer (2018), examining university students' awareness of climate change and environmental problems, it was found that there was a positive and moderately significant relationship between students' perceptions/concerns about climate change and their attitudes toward environmental problems and that as students' attitudes toward environmental problems increased, their perceptions of climate change also increased. Tümer, İpek, and Ercan (2024) examined the awareness, anxiety, and hope levels of nursing students regarding climate change; it was determined that the awareness and anxiety of nursing students regarding climate change positively affected the hope for the prevention of climate change, and initiatives to increase climate change awareness could mobilize nursing students.

In a study examining the attitudes of nurse academics toward climate change and environmental awareness, it was found that academic nurses had knowledge about climate change, exhibited some practices/behaviors in their own lives, but did not emphasize the importance of the subject in their courses (Dündar and Özsoy 2020).

## 5 Conclusions

It is also suggested that the knowledge and awareness levels of nursing students regarding climate change and environmental problems are insufficient, and that climate change and its inevitable effects on the environment and its direct/indirect effects on health should be included in the curriculum of the nursing department (Küçük Biçer and Vaizoğlu 2015; Fırat Kılıç et al. 2024). As a result, in this study, it was found that nursing students were more sensitive to environmental health as their anxiety levels about climate change increased. In this context, it is recommended that students should have knowledge and awareness about climate change in undergraduate nursing education, and a course content related to the subject should be included in the undergraduate curriculum. In addition, it is critical to ensure that nursing students understand their personal roles in defending the health system on climate change and environmental health, their roles in direct patient care, community education, and health policy development at a time when they have not yet started their profession (Anåker et al. 2021).

## 6 Recommendations for Further Research

Nurses play a crucial role in addressing the impact of climate change on public health. They are front‐line healthcare professionals who are positioned to identify and address the impact climate change on individuals and communities. Environmental health is an important concept for community health nursing. Nurses have important roles in developing and protecting health. For this reason, it is recommended to be aware of global health problems, the impact of these problems on human and environmental health, and to conduct research on all variables that will affect this field. Qualitative studies should be conducted in order to clearly understand the views of nursing students on climate change and environmental sensitivity. Interventional studies should be carried out to increase students' awareness of climate change and the environment, and studies should be carried out to examine whether there is an increase in students' awareness at grade level.

## 7 Strengths and Limitations

The fact that environmental problems have recently reached serious dimensions in the world and have become a significant threat to public health makes it obligatory for all health professionals to be sensitive to the environment. The fact that the environmental sensitivity and climate change concern of nursing students, who are in constant contact with healthy and sick individuals both in the field and in the hospital environment and who assume an important role in protecting and improving public health, have been examined with this study, especially the fact that the study is a pioneer in this field in Turkey is one of the strengths of our study. It is important to raise awareness of two important concepts such as climate change and environmental sensitivity in nursing students before they start their profession and to draw attention to this issue. The limitations of this study are that the students participating in the study were selected only from volunteers and answered the questions according to their reports and that just nursing students were included in the study. The study was conducted with a cross‐sectional research design. Additionally, all data collection tools were answered through the students’ self‐reports. This was considered as a limitation since the responses, including attitudes, may change over time.

## Disclosure

The authors alone are responsible for the content and writing of the article.

## Conflicts of Interest

The authors declare no conflicts of interest.

## Data Availability

Data sharing not applicable to this article as no datasets were generated or analyzed during the current study.
